# Successful Treatment of Refractory Palmoplantar Pustulosis With Bimekizumab in a Patient With Multiple Sclerosis: A Case Report

**DOI:** 10.7759/cureus.107090

**Published:** 2026-04-15

**Authors:** Osman Mahboob, John Acosta-Peñaloza, Brittani Kongala, Yusuf Amawi, Cynthia Tie

**Affiliations:** 1 Clinical Sciences, Florida State University College of Medicine, Tallahassee, USA; 2 Dermatology, Family Dermatology of North Florida, Tallahassee, USA

**Keywords:** bimekizumab, interleukin 17a/f, multiple sclerosis, palmoplantar pustulosis, psoriasis

## Abstract

We present a case of refractory palmoplantar pustulosis (PPP) in a female in her early 50s with a history of well-controlled multiple sclerosis (MS). PPP is a dermatological condition characterized by chronic pustules often accompanied by fissures on the palms of the hands or soles of the feet. It is often seen in association with psoriasis vulgaris and arthritis; however, it can also present as isolated lesions. Although several treatment options exist for PPP, including topical corticosteroids, topical calcineurin inhibitors, vitamin D analogs, narrowband ultraviolet B (NBUVB) phototherapy, biologics, and topical retinoids, PPP remains a complex disease to treat.

In this case, the patient failed to achieve significant improvement despite trialing multiple therapies, including topical corticosteroids, topical agents, systemic therapy, NBUVB phototherapy, and several biologics, as well as adjunctive antifungal therapy, with persistent pain secondary to fissuring and scaling of her plantar skin.

Due to the refractory nature of her PPP, bimekizumab, an anti-IL-17A and IL-17F antibody therapy, was administered as a subcutaneous injection at weeks 0, 4, 8, 12, and 16, followed by maintenance dosing every eight weeks. The patient experienced a significant and sustained improvement in her PPP.

Although bimekizumab is primarily used to treat psoriasis and psoriatic arthritis, this case highlights its potential role in the treatment of refractory PPP. There are concerns regarding the safety of biologic treatment in patients with MS due to the possibility of worsening MS symptoms. However, these concerns are primarily associated with anti-tumor necrosis factor therapies and may not be directly applicable to IL-17 inhibitors such as bimekizumab. This clinical case suggests that bimekizumab may be a reasonable therapeutic option in patients with well-controlled MS, though further studies are needed to better characterize its safety in this population.

## Introduction

Palmoplantar pustulosis (PPP) is a chronic inflammatory dermatosis characterized by sterile pustules, erythema, hyperkeratosis, and fissuring localized to the palms and soles [1,2]. The disease course is often relapsing and refractory, resulting in substantial physical discomfort, functional impairment, and psychosocial burden.

PPP is frequently resistant to conventional therapies, including high-potency topical corticosteroids, vitamin D analogs, topical calcineurin inhibitors, phototherapy, biologics, and systemic agents such as retinoids and small-molecule immunomodulators [1,2]. As a result, effective long-term management remains challenging, and many patients experience persistent disease despite multiple treatment modalities.

Recent advances in the understanding of PPP pathophysiology have highlighted the central role of the IL-17 pathway, particularly IL-17A and IL-17F, in driving keratinocyte activation and neutrophil recruitment within lesional skin [3,4]. The use of biologic therapies targeting immune pathways has raised safety considerations in patients with autoimmune neurologic disease, as certain immunomodulatory agents have been associated with disease exacerbation or demyelinating events [5].

Although historically classified as a variant of pustular psoriasis, PPP is increasingly recognized as a distinct clinical entity with unique epidemiologic, genetic, and immunologic features [2,6]. While IL-17A inhibitors, such as secukinumab and ixekizumab, have demonstrated efficacy in plaque psoriasis, their effectiveness in PPP has been variable, suggesting that broader cytokine blockade may be required in refractory disease [4,7].

Bimekizumab is a humanized IgG1 monoclonal antibody that selectively inhibits both IL-17A and IL-17F and is currently approved for the treatment of psoriasis and psoriatic arthritis [3,4]. Emerging evidence suggests that dual IL-17A/F inhibition may offer therapeutic benefit in pustular and palmoplantar variants of psoriasis, including PPP, by providing more complete suppression of IL-17-driven inflammation, leading to improved lesion clearance, reduction in pustule formation, and more rapid and sustained clinical responses compared to IL-17A inhibition alone [1,7,8]. Data regarding the safety of IL-17 pathway inhibition in patients with multiple sclerosis (MS) remain limited, though early clinical studies have explored the neurologic effects of IL-17A blockade without clear evidence of disease worsening [9].

Here, we report a case of refractory PPP in a patient with well-controlled MS who experienced significant clinical improvement following treatment with bimekizumab.

## Case presentation

**Figure 1:**
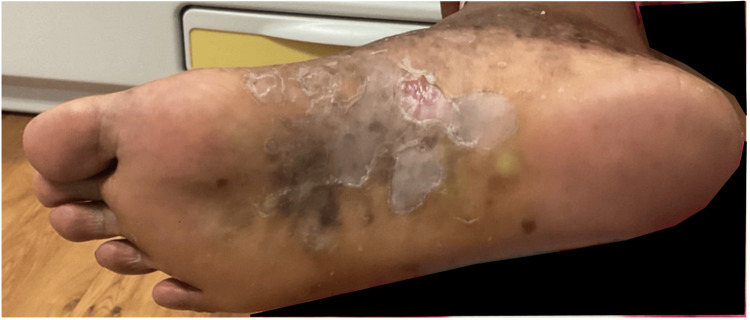
Plantar surface of patient's right foot, demonstrating persistent pustules, thick scaling, and fissures prior to bimekizumab treatment.

A female in her early 50s with a five-year history of PPP presented with persistent pustules, thick scaling, and fissures (Figure 1).

This was associated with significant pain and discomfort localized to the right plantar surface. No other areas of the body were affected. Despite treatment with multiple therapies over several years, symptoms persisted, significantly impairing daily activities. Prior treatments included topical corticosteroids, with triamcinolone initiated in March 2020 and clobetasol initiated in September 2021, as well as topical agents including crisaborole (initiated March 2020), ruxolitinib cream (April 2023 to September 2023), and halobetasol and tazarotene combination therapy (June 2023 to September 2023). Systemic therapy with apremilast was administered from September 2020 to October 2021, and acitretin was initiated in 2024 but discontinued in April 2024 due to worsening joint pain and MS symptoms.

Phototherapy with NBUVB was used from October 2020 to March 2022 and was later restarted in April 2024. The patient also received multiple biologic therapies, including secukinumab (October 2021 to March 2022), certolizumab pegol (March 2022 to March 2023), ixekizumab (April 2023 to August 2023), and dupilumab (November 2023 to March 2024). Adjunctive treatments included regular emollient use (June 2023 to present).

The patient’s medical history was notable for MS, well-controlled with Ofatumumab 20 mg/0.4 mL subcutaneous injections. At the time of presentation, the patient was also undergoing treatment for tinea pedis, effectively managed with terbinafine 250 mg twice daily for seven days, repeated monthly. Additional comorbidities included alcohol use (less than one drink per day) and active daily tobacco use, which is strongly associated with PPP. She reported an allergy to amoxicillin.

On examination, the right plantar surface exhibited thick, hyperkeratotic plaques and subcutaneous purulent collections, with no involvement of the hands or other areas. Given the localized involvement of the plantar surface, body surface area estimation is limited (2%-3%); however, disease severity was assessed clinically with a Psoriasis Physician Global Assessment (PGA) score of 3.0. Partial improvement was observed with NBUVB phototherapy, administered three times weekly, with mild reduction in scaling and erythema; however, pustules and fissuring persisted. Acitretin was discontinued two weeks prior due to exacerbation of joint pain and MS symptoms, with improvement in symptoms shortly after withdrawal. 

Given the refractory nature of her PPP and prior treatment failures, bimekizumab, a novel IL-17A and IL-17F inhibitor, was initiated. The treatment regimen followed standard dosing guidelines, consisting of 320 mg subcutaneous injections at weeks 0, 4, 8, 12, and 16, followed by maintenance dosing every eight weeks, with no deviations from the established protocol. Baseline laboratory testing, including CBC, CMP, and a QuantiFERON Gold test, was within normal limits, and the patient was cleared for treatment.

**Figure 2:**
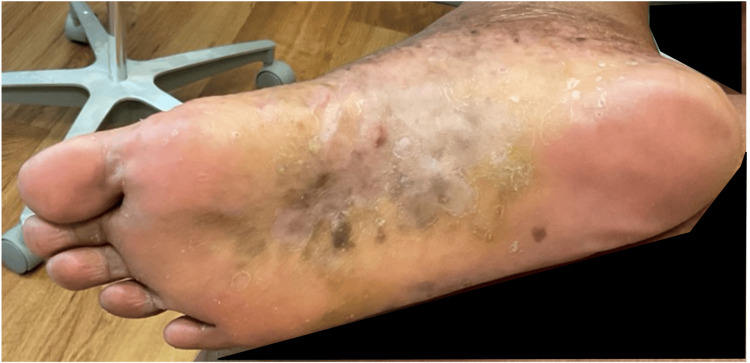
Plantar surface of the patient's right foot six weeks after beginning treatment with bimekizumab, demonstrating significant improvement of fissuring and scaling.

A substantial reduction in BSA involvement was noted at the one-month follow-up, decreasing to 1.0%. However, the PGA score remained at 3.0, likely reflecting persistent residual disease despite improvement in scaling and pustules. By the six-week follow-up, the plaques on the right foot had thinned significantly, and no deep-seated pustules were observed (Figure 2).

The patient reported that the affected area was no longer swollen or tender to palpation, with corresponding improvement in pain and daily functional activities. Although the treatment goal had not yet been fully achieved, these findings indicated meaningful clinical improvement. No adverse effects or complications were reported during the treatment course with bimekizumab; no clinical evidence of MS exacerbation was observed during the treatment period. The patient continued therapy beyond this time point; however, long-term follow-up data beyond the six-week time point were not available.

## Discussion

PPP is a chronic inflammatory dermatosis affecting the palms and soles that is frequently refractory to conventional topical and systemic therapies [1,2]. Although historically grouped with pustular psoriasis, PPP is increasingly recognized as a distinct clinical entity with unique immunologic features, including prominent neutrophilic infiltration and dysregulation of the IL-17 pathway [2,6,7]. These features may contribute to the limited and inconsistent response observed with therapies effective in plaque psoriasis.

Both IL-17A and IL-17F play central roles in PPP pathogenesis by promoting keratinocyte activation and neutrophil recruitment; however, IL-17F is expressed at substantially higher concentrations in inflamed tissue and may sustain chronic inflammation despite IL-17A blockade alone [3,4,7]. This distinction provides a biologic rationale for dual IL-17A and IL-17F inhibition in refractory disease. Bimekizumab, a monoclonal antibody targeting both cytokines, therefore offers a mechanistically broader approach compared with IL-17A-only inhibitors such as secukinumab and ixekizumab [3,4].

The present case underscores the therapeutic challenges associated with PPP. Despite prior treatment with multiple topical agents, phototherapy, systemic retinoids, and several biologic therapies, including IL-17A inhibitors, the patient experienced persistent, functionally limiting disease. Following initiation of bimekizumab, the patient demonstrated a rapid and clinically meaningful reduction in body surface area involvement, thinning of hyperkeratotic plaques, and resolution of deep-seated pustules. While complete clearance was not achieved within the follow-up period, the observed improvement suggests that dual IL-17A/F inhibition may be effective in select patients with treatment-resistant PPP.

The coexistence of MS introduces additional considerations when selecting biologic therapy. Certain immunomodulatory agents, particularly tumor necrosis factor-α inhibitors, have been associated with demyelinating events and are generally avoided in patients with MS [5,9]. Although IL-17 signaling has been implicated in MS pathophysiology, available clinical data regarding IL-17 inhibition in this population remain limited and mixed [9,10]. In this case, the patient’s MS remained stable throughout bimekizumab therapy, with no clinical evidence of disease exacerbation, suggesting that bimekizumab may be cautiously considered in patients with well-controlled MS under close monitoring.

PPP is associated with substantial morbidity, including pain, fissuring, impaired ambulation, and reduced quality of life [2,6]. The patient’s reported improvement in swelling, tenderness, and functional discomfort following bimekizumab treatment likely translated into a meaningful quality of life benefit, even in the absence of complete lesion resolution. The patient was counseled on smoking cessation, given its known association with PPP; however, she was not willing to commit to cessation at this time [11].

This case report is limited by its single-patient design and relatively short duration of follow-up, which prevents conclusions regarding sustained remission or long-term neurologic safety. Nonetheless, it adds to the emerging evidence supporting bimekizumab as a potential therapeutic option for refractory PPP and provides limited preliminary data regarding its neurologic safety in patients with coexisting MS. Larger prospective studies and long-term observational data are needed to better define the durability of response, safety profile, and optimal patient selection for dual IL-17A/F inhibition in PPP.

## Conclusions

This case highlights the potential role of bimekizumab as a treatment option for refractory PPP, including in patients with complex medical histories such as MS. In a patient with long-standing, treatment-resistant disease, dual IL-17A and IL-17F inhibition was associated with meaningful clinical improvement without evidence of neurologic disease exacerbation.

While the findings from this single case should be interpreted with caution, they support further investigation into the use of bimekizumab for refractory PPP, particularly in patient populations traditionally considered challenging for biologic therapy. Additional studies are needed to better define long-term efficacy, safety, and patient selection criteria before broader conclusions can be drawn.
